# Multi-isotopic (*δ*2H, *δ*13C, *δ*15N) tracing of molt origin for European starlings associated with U.S. dairies and feedlots

**DOI:** 10.1371/journal.pone.0237137

**Published:** 2020-08-10

**Authors:** Scott J. Werner, Justin W. Fischer, Keith A. Hobson

**Affiliations:** 1 United States Department of Agriculture, Animal and Plant Health Inspection Service, Wildlife Services, National Wildlife Research Center, Fort Collins, CO, United States of America; 2 Department of Biology and Environment and Climate Change Canada, University of Western Ontario, London, ON, Canada; Senckenberg Gesellschaft fur Naturforschung, GERMANY

## Abstract

Introduced bird species can become invasive in agroecosystems and their management is inhibited if their origin and movements are not well understood. Stable isotope measurements of feathers can be used to infer molt origins and interstate movements in North America. We analyzed stable-hydrogen (*δ*^2^H), carbon (*δ*^13^C) and nitrogen (*δ* ^15^N) isotope ratios in feathers to better understand the molt origin of European starlings (*Sturnus vulgaris*) collected at dairies and feedlots throughout the United States. Primary feathers were used from 596 adult and 90 juvenile starlings collected during winter at dairies and feedlots that experience starling damages in Arizona, California, Colorado, Idaho, Illinois, Iowa, Kansas, Minnesota, Missouri, Nebraska, Nevada, New Hampshire, New Mexico, New York, North Carolina, Oregon, Texas, Vermont, Washington and Wisconsin. The best-fit model indicated that the combination of feather *δ*^2^H, *δ*^13^C and *δ*^15^N values best predicted the state where samples were collected and thus supported use of this approach for tracing molt origins in European starlings. Interestingly, molt origins of starlings collected at dairies and feedlots generally west of ˗90° longitude (i.e. 11 of 15 states west of the Mississippi River, including Wisconsin) were assigned to the collection state and/or the state adjacent to the collection state. In contrast, molt origins of starlings collected generally east of ˗90° longitude (four of five eastern states) were not assigned to the collection state and/or the state adjacent to the collection state. Among all starlings (*N* = 686), 23% were assigned to the collection state and 19% were assigned to the state adjacent to the collection state. Among all males (*N* = 489) and all females (*N* = 197), 23% and 26% were assigned to the collection state and 19% and 13% were assigned to the state adjacent to the collection state, respectively. We observed a greater proportion (88%) of juvenile starlings assigned to states other than their collection state (i.e. potentially a result of natal dispersal) than that proportion (76%) in adult starlings. This study included an unprecedented sample of feather isotopes from European starlings throughout the United States. As a novel contribution to the ecology and management of invasive and migratory passerines, we demonstrate how such feather isoscapes can be used to predict molt origin and, potentially, interstate movements of European starlings for subsequent ecological and management investigations.

## Introduction

Dispersal and movements of migratory birds on breeding and non-breeding grounds are fundamental to planning conservation and management efforts, both nationally and internationally [1]. Overabundant species, invasive species and agricultural depredation caused by some wild birds pose considerable management challenges [2]. One hundred European starlings (*Sturnus vulgaris*) from Europe were released in New York City’s Central Park in 1890–1891. European starlings have since flourished in the U.S. and starlings are currently year-round residents throughout most of North America [3, 4]. Westward colonization of European starlings ranged to approximately ˗103° longitude in 1937, including eastern South Dakota, eastern Nebraska, eastern Colorado, and northern Texas [5]. Starlings were reported in California in 1942 [6], Oregon and Washington in 1943 [7, 8], British Columbia in 1947 [9] and Alaska in 1952 [3]. In North America, the European starling is an invasive species known to cause considerable damage to agricultural industries, especially those related to the production of fruit, beef and milk [10–12]. Indeed, by 1934 “certain undesirable traits of the species had raised the question of the possibility of controlling its numbers, and the first step in the scientific control of an organism is the careful study of its habits and its movements” [13].

Rapid colonization of North America by European starlings and extension of their breeding range resulted from migrations and wanderings of first-year and non-breeding second-year individuals [3, 4]. Natal dispersal, in particular, enables exchange of individuals among established populations and colonization of new areas [14]. High density of local, non-migratory populations (which limits genetic drift) and high potential for juvenile dispersal (which blends local populations) has resulted in a North American population of European starlings which is genetically homogeneous across its range [14]. Consistent with this species’ rapid expansion history, reduced-representation genome sequencing (ddRADseq) of starlings collected throughout the U.S. illustrated low genome-wide differentiation and few outliers of population differentiation due to genetic structure (i.e. F_ST_ fixation index) [15]. Banding and allozyme allele frequency data suggest that the North American population of European starlings is nearly a panmictic, or random-mating population [14]. This panmictic habit is likely influenced by irregular movements and apparent random selection of breeding sites by after-hatching-year (AHY) starlings [3].

European starlings are partial migrants in North America [3, 13]. The proportion of migratory and non-migratory starlings likely varies with geographic location and season [3]. On an annual basis, more migratory starlings occur in central and southern Midwestern states than in Great Lakes states, the northeast and Atlantic seaboard of the U.S. [3]. These migratory trends are perhaps due to 1) starlings being more migratory on the edge of their range or within low-density populations and 2) the fact that areas with highest out-of-state banding recoveries are close to main flyways of starling migration (i.e. from Ontario through Ohio, along Ohio and Mississippi Rivers) [3].

Several lethal and non-lethal control programs for European starlings have been investigated in the U.S. [2], but without an understanding of the movements and dispersal of starlings, it is not clear if individuals are being recruited from other (e.g. non-control) sites and whether localized damage management is an effective solution. Describing movements of abundant species across state boundaries, where control measures may vary, has hitherto been virtually impossible based on conventional tracking technologies [16]. The measurement of intrinsic markers, such as abundance of naturally-occurring stable isotopes, has emerged as an effective tool in assigning migratory birds to their molt origin [17] in particular and in investigating fundamental questions regarding invasion ecology [18], in general.

The primary objective of this study was to use *δ*^2^H, *δ*^13^C and *δ*^15^N measurements in feathers of European starlings collected at U.S. dairies and feedlots to better understand their molt origin and interstate movements. We expected *δ*^2^H and *δ*^13^C measurements to provide greatest resolution for spatial assignments but included *δ*^15^N analyses because this isotope is also sensitive to land-use practices and other anthropogenic factors that may vary across our study area [1, 19, 20]. We included each of *δ*^2^H, *δ*^13^C and *δ* ^15^N measurements because previous studies have demonstrated best-fit modeling associated with a multi-isotopic approach to the analysis of feather isotopes in pest birds [1].

Little is known of the habits of immature starlings before their first breeding attempt, probably owing to difficulty in distinguishing between immature and adult starlings after the prebasic molt is finished in September or October [4]. Some immature birds may disperse and wander extensively during summer but the extent of this movement is poorly known [3, 4]. Our secondary objectives were to comparatively investigate sex-specific movements of males and females, and age-specific movements of juvenile and adult starlings associated with U.S. dairies and feedlots. We also investigated relationships of predicted precipitation *δ*^2^H and feather *δ*^2^H, and *δ*^13^C and *δ*^15^N values in starling feathers and agricultural crops.

We collected adult and juvenile starlings [4, 21] in winter to investigate their putative molt or natal origins. These fundamental, yet previously-unknown spatial relationships can be used to prescribe and implement management efforts for invasive species, overabundant species and wild birds associated with U.S. dairies and feedlots, including European starlings. The spatial extent of starling research and management regarding agricultural depredation, however, is usually defined by state or provincial boundaries. We were therefore interested to use stable isotopes to investigate molt origins and interstate movements, but fully recognize that isotopic patterns may not follow state or provincial boundaries. Nonetheless, we hypothesized that there are spatial and age-specific patterns in the movements of European starlings associated with U.S. dairies and feedlots. Starling populations south of 40° latitude exhibited little migration in winter away from their nesting location [22]. We therefore predicted that starlings collected south of 40° latitude would primarily be assigned to their collection state. We predicted no differences in molt origin or interstate movements between male and female starlings because sexually monomorphic species such as European starlings are expected to exhibit no sex-specific differences in migration distances [22]. We further predicted that dispersal of juveniles would exceed interstate movements of adult starlings collected at U.S. dairies and feedlots.

## Methods

### Feather sampling

**Fig 1:**
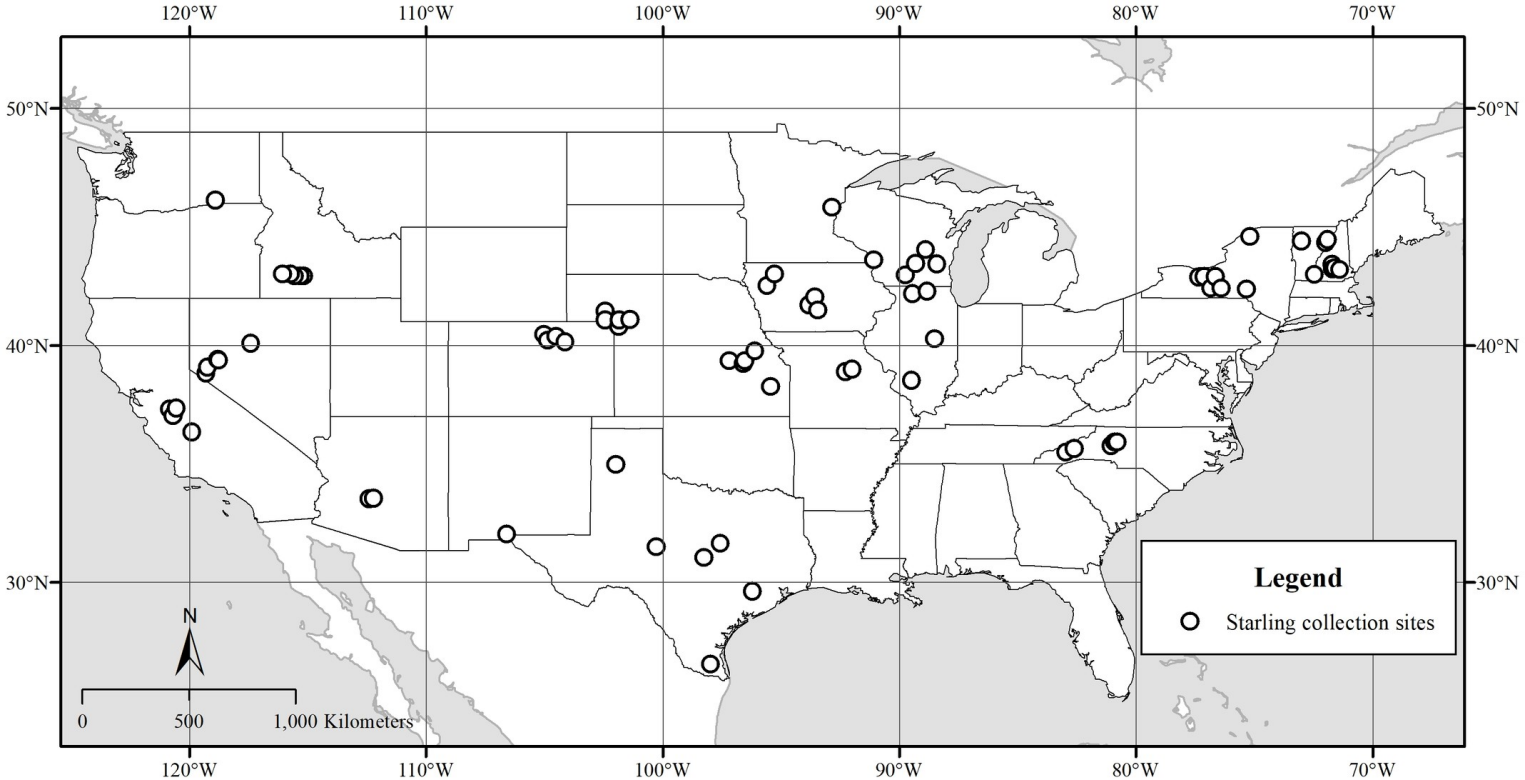
Geographic range of feather-sample collection sites used to develop multi-isotopic depictions of molt origin for European starlings collected at U.S. dairies and feedlots.

We opportunistically collected 596 adult and 90 juvenile starlings during winter (January–March 2016 and January–February 2017) at U.S. dairies and feedlots that experience starling damages. For this study, we selected the 20 states most commonly associated with starling damage management provided by the United States Department of Agriculture’s (USDA) Wildlife Services program, including Arizona, California, Colorado, Idaho, Illinois, Iowa, Kansas, Minnesota, Missouri, Nebraska, Nevada, New Hampshire, New Mexico, New York, North Carolina, Oregon, Texas, Vermont, Washington and Wisconsin. We planned collections for 5–10 birds per site and 2–5 sites per state. Collection sites were selected ≥5 km apart to maximize the geographic scope of our study. USDA’s Wildlife Services personnel used a handheld GPS unit to record the latitude and longitude of each collection site for subsequent spatial analyses. Because our sample collection sites were U.S. dairies and feedlots associated with previous and/or ongoing starling damage management by USDA’s Wildlife Services program, the spatial distribution of our samples (i.e. within sampled states) and our sampled states (nationally) was non-random and non-uniform (Fig 1).

Starlings were collected using lethal gunshot, avicide or live traps. Live-trapped starlings were subsequently euthanized using procedures approved by the American Veterinary Medical Association (e.g. CO_2_; [23]). Where necessary, bird collections were authorized by state scientific collection licenses. Because European starlings are invasive to the U.S., no federal scientific collecting permit was necessary for these collections. The collection and use of starlings for this study were approved by USDA National Wildlife Research Center’s Institutional Animal Care and Use Committee (QA-2572, S.J. Werner- Study Director).

We removed one wing from each collected bird. Wing samples were stored in labeled paper bags and frozen until stable isotope analyses. We removed a single outer primary (P8 or P9) from each wing for stable isotope analysis. Primary feathers of European starlings are molted in late June–September (especially July–early September) [4]. Our stable isotope data for each site are based upon single *δ*^2^H, *δ*^13^C and *δ*^15^N values for each individual bird collected at that site. For the purposes of this paper, we refer to starlings that were born in the calendar year prior to the collection year as juveniles and those that were at least one year old in that year as adults. We recognize that the terms second-year (SY) and after-second-year (ASY) also apply in this context.

### Stable isotope analysis

All feathers were cleaned of surface oils in 2:1 chloroform:methanol solvent rinse and prepared for *δ*^2^H, *δ*^13^C and *δ*^15^N analyses at the University of Western Ontario. The *δ*^2^H value of non-exchangeable hydrogen in feathers was determined using previously-described methods and two calibrated keratin hydrogen-isotope reference materials (CBS: -197.‰, KHS: -54.1 ‰) in keeping with the comparative equilibrium approach [24]. Hydrogen isotopic measurements were performed on H_2_ gas derived from high-temperature (1350°C) flash pyrolysis of 350 ± 10 μg feather subsamples and keratin standards using continuous-flow isotope-ratio mass spectrometry. Measurements of the two keratin laboratory reference materials, corrected for linear instrumental drift, were both accurate and precise with typical within-run (*n* = 5) SD values of <2 ‰. All results are reported for non-exchangeable H expressed in typical delta (*δ*) notation, in units per mil (‰), and normalized on the Vienna Standard Mean Ocean Water–Standard Light Antarctic Precipitation (VSMOW-SLAP) standard scale.

For *δ*^13^C and *δ*^15^N analyses, 0.5–1.0 mg of feather material was combusted online using a Eurovector 3000 (Milan, Italy– www.eurovector.it) elemental analyzer. Resulting CO_2_ was separated by gas chromatography (GC) and introduced into a Nu Horizon (Nu Instruments, Wrexham, UK– www.nu-ins.com) triple-collector isotope-ratio mass-spectrometer via an open split and compared to a pure CO_2_ or N_2_ reference gas. Stable nitrogen (^15^N/^14^N) and carbon (^13^C/^12^C) isotope ratios were expressed in *δ* notation, as parts per thousand (‰) deviation from primary standards, atmospheric AIR and Vienna Pee Dee Belemnite (VPDB). Using previously calibrated internal laboratory standards (powdered keratin [BWB II: *δ* ^13^C = -20.0*‰*, *δ*^15^N = -14.1‰ and gelatin: *δ* ^13^C = -13.6*‰*, *δ*^15^N = -4.7‰]) within run (*n* = 5), precision for *δ*^15^N and *δ*^13^C measurements was ~ ± 0.15‰. Our master dataset (feather isotopes for each of 686 European starlings with collection-site location data) has been made publically available for subsequent investigations (S1 Table).

### Statistical analysis

We used an analysis of variance (PROC GLM, SAS v9.4) and linear regression to analyze stable isotopes among states. We used Tukey-Kramer multiple comparisons to separate mean stable isotopes among states. For the primary objective of this study regarding molt origin and interstate movements of European starlings collected at U.S. dairies and feedlots, we used discriminant function analysis (SAS v9.4) to assign states of molt origin from feather *δ*^2^H, *δ*^13^C and *δ*^15^N values. For each feather sample, a predicted location of molt origin was assigned to the collection state (i.e. the state in which the sample was collected), the state adjacent to the collection state, or other state (not collection state or state adjacent to collection state). The assignment of states associated with molt origin may therefore be affected by juxtaposition of collection states and proximity of collection sites to state boundaries.

The STEPDISC procedure of SAS was used to identify the best-fit model among all possible combinations of *δ*^2^H, *δ*^13^C and *δ*^15^N data. We used descriptive statistics (mean, SE, min, max, lower and upper confidence limits) to summarize stable isotopes among states. We used log-transformed percentages and a kriged surface (ArcGIS v 10.6 Geostatistical Analyst ^TM^ extension; ESRI, Redlands, CA) to illustrate the national pattern of collection-state assignments among all starlings (*N* = 686). Linear regression was used to examine the relationship between feather *δ*^2^H values and the amount-weighted mean annual precipitation (MAD) or growing-season average (GSD) *δ*^2^H values predicted for collections sites.

### Relationship of predicted precipitation *δ*^2^H and feather *δ*^2^H

In addition to our discriminant function analysis approach using all three stable isotope measurements, we also examined the starling dataset for evidence of immigration using feather δ^2^H measurements alone. There have been several approaches previously used to derive an estimate of local vs. non-local origins of birds using feather δ^2^H measurements and comparing those to values expected for a given location based on the long-term Global Network of Isotopes in Precipitation (GNIP; https://www.iaea.org/services/networks/gnip). Most recently, López Calderón et al. [25] recommended an approach which applied the normal probability density function to ascertain the probability of a feather being molted at a particular site given the expected distribution of feather δ^2^H values for that site. However, in our case, we did not have a starling-specific δ^2^H_f_ vs. δ^2^H_p_ calibration curve for known-origin starlings and we recognize that this relationship can differ between juvenile and adult birds during their post-natal and post breeding molt [26]. Instead we used the δ^2^H_f_ vs. δ^2^H_p_ relationship for juvenile and adult starlings separately and derived the SD of residuals with the assumption that residual δ^2^H_f_ variance would be related to the degree of dispersal.

### Relationship of *δ*^13^C and *δ*^15^N values with agricultural crops

It is well recognized that *δ*^13^C values in animal tissues can provide insight into relative proportions of C3 vs. C4 carbon at the base of the foodweb supporting those animals. Corn is a C4 plant and its contribution to the carbon budget of consumers is readily distinguishable isotopically in an otherwise largely C3 biome. In our case, starlings could consume corn directly from crops [27] or more indirectly from foods fed to cows and cattle at dairy farms and feedlots, respectively [11, 12]. Our objective here was to obtain a simple estimate of percent C4 in the diet of starlings during feather formation. We assumed this would be directly related to the proportion of corn in diets of starlings and largely reflect their dependence on agricultural based products. Following Werner et al. [1], we assumed that a starling feeding on a 100% C3 diet would have a feather *δ*^13^C value of -26‰ and one feeding on a 100% C4 diet would have a feather *δ*^13^C value of -11‰. This allowed a simple two-endpoint, one isotope mixing model to estimate percent C4 in the diet.

Interpreting feather δ^15^N values is more challenging as nitrogen in foodwebs is influenced by several natural and anthropogenic processes. In agricultural settings, use of organic or inorganic fertilizer has a significant effect on local foodwebs as will general geographic location related to NOx fallout and relative forest cover [19]. Our approach here was to examine δ^15^N data post-hoc but we had no strong a priori expectations.

## Results

### Interstate comparison of feather isotopes

We observed a considerable range in *δ*^2^H (˗146.8 to ˗21.6 ‰), *δ*^13^C (˗26.0 to ˗11.1 ‰) and *δ*^15^N (6.3 to 24.3 ‰) values in feathers of all European starlings (*N* = 686; Table 1). The range of latitude and longitude among collection sites was 26.548–46.137° N and 71.382–120.833° W (Fig 1). We observed greatest differences among states in *δ*^2^H_f_ values. Stable hydrogen isotopes were greatest in feathers of starlings collected in Texas (Table 1). Non-overlapping, 95% confidence intervals suggested that *δ*^2^H_f_ values were similar among feathers collected in North Carolina and New Mexico; New Mexico, Kansas, Missouri, Illinois, Arizona, Iowa, Wisconsin, New York, New Hampshire, California and Minnesota; Wisconsin, New York, New Hampshire, California, Minnesota, Vermont, Colorado and Nebraska; and Nevada, Washington, Idaho and Oregon (Table 1).

**Table 1:** Interstate comparisons of feather isotopes in all European starlings (*N* = 686) collected at U.S. dairies and feedlots. For each of *δ*^2^H, *δ*^13^C and *δ*^15^N, statistically different means between collection states are indicated by unique letters in the right column.

Isotope/state	Mean	SE	*n*	Min.	Max.	Lower CL	Upper CL	Tukey-Kramer *P* < 0.05
*δ*^2^H								
Texas	-43.1	1.1	62	-67.4	-21.6	-45.4	-40.9	a
North Carolina	-52.9	2.1	25	-84.4	-38.8	-57.0	-48.8	b
New Mexico	-65.0	4.3	9	-97.2	-53.7	-73.6	-56.4	b,c
Kansas	-65.6	2.1	41	-92.4	-42.7	-69.7	-61.5	c
Missouri	-66.3	2.8	19	-91.2	-52.2	-71.9	-60.6	c
Illinois	-71.3	1.7	42	-99.2	-49.5	-74.6	-68.0	c
Arizona	-71.5	1.1	60	-89.6	-54.9	-73.6	-69.4	c
Iowa	-72.9	1.1	50	-89.9	-56.6	-75.0	-70.8	c
Wisconsin	-76.9	1.3	49	-109.9	-53.9	-79.5	-74.3	c,d
New York	-77.1	1.1	68	-101.8	-57.2	-79.2	-74.9	c,d
New Hampshire	-77.5	1.9	32	-101.2	-58.5	-81.2	-73.8	c,d
California	-79.2	4.5	20	-146.8	-53.5	-88.2	-70.1	c,d
Minnesota	-84.7	4.1	4	-93.1	-75.8	-92.8	-76.6	c,d
Vermont	-86.5	2.3	17	-104.9	-68.4	-91.1	-81.9	d
Colorado	-95.1	1.6	46	-125.3	-70.3	-98.2	-92.0	d
Nebraska	-95.1	2.9	25	-121.8	-64.4	-100.9	-89.4	d
Nevada	-106.3	1.9	71	-139.9	-64.3	-110.1	-102.5	e
Washington	-107.6	3.3	15	-133.7	-88.3	-114.2	-100.9	e
Idaho	-115.5	1.6	26	-134.8	-98.1	-118.7	-112.3	e
Oregon	-119.4	8.8	5	-136.6	-94.7	-137.0	-101.7	e
*δ*^13^C								
Texas	-17.4	0.4	62	-23.2	-11.1	-18.2	-16.7	a
California	-19.1	0.6	20	-24.5	-14.3	-20.4	-17.9	a,b
New Mexico	-19.2	0.5	9	-22.5	-17.0	-20.3	-18.2	a,b,c
North Carolina	-19.5	0.5	25	-24.1	-15.8	-20.5	-18.5	b
Arizona	-19.6	0.2	60	-22.6	-12.7	-20.1	-19.2	b
New Hampshire	-20.2	0.6	32	-24.5	-12.3	-21.3	-19.1	b,c
Kansas	-20.7	0.4	41	-23.9	-15.0	-21.4	-20.0	b,c
Iowa	-21.1	0.3	50	-24.0	-14.9	-21.7	-20.5	b,c
Missouri	-21.3	0.4	19	-23.7	-17.8	-22.2	-20.5	b,c
Vermont	-21.8	0.6	17	-24.8	-16.5	-23.0	-20.5	b,c
Illinois	-21.8	0.3	42	-25.1	-16.7	-22.4	-21.1	c
Colorado	-21.9	0.3	46	-26.0	-16.2	-22.4	-21.3	c
Nebraska	-21.9	0.5	25	-25.8	-15.0	-23.0	-20.8	c
Washington	-22.0	0.2	15	-24.1	-20.9	-22.5	-21.6	b,c
Nevada	-22.1	0.2	71	-25.7	-12.7	-22.5	-21.6	c
Oregon	-22.2	0.7	5	-23.5	-19.8	-23.5	-20.8	b,c
New York	-22.2	0.3	68	-25.1	-16.0	-22.8	-21.7	c
Wisconsin	-22.3	0.2	49	-25.0	-18.5	-22.7	-21.9	c
Idaho	-22.8	0.1	26	-24.8	-21.1	-23.1	-22.5	c
Minnesota	-23.6	0.3	4	-24.1	-22.8	-24.2	-22.9	b,c
*δ*^15^N								
Texas	13.5	0.4	62	9.2	24.3	12.8	14.2	a
New Mexico	13.4	0.6	9	10.2	16.9	12.2	14.6	a,b
Colorado	12.8	0.4	46	7.3	17.7	12.1	13.6	a,b
California	12.4	0.6	20	6.7	17.2	11.3	13.6	a,b
Illinois	11.7	0.4	42	8.1	18.2	10.8	12.5	b
Iowa	11.5	0.2	50	8.1	15.5	11.0	12.0	b
North Carolina	11.4	0.6	25	6.8	16.6	10.1	12.7	b,c
Vermont	11.4	0.5	17	8.3	14.7	10.4	12.3	b,c
Missouri	11.3	0.5	19	8.2	14.5	10.4	12.2	b,c
Nebraska	11.3	0.5	25	6.5	15.8	10.3	12.3	b,c
Oregon	11.1	0.7	5	9.6	13.8	9.7	12.6	a,b,c
Arizona	11.0	0.2	60	7.8	16.2	10.6	11.4	b,c
Idaho	10.9	0.4	26	7.6	15.9	10.1	11.6	b,c
Kansas	10.8	0.3	41	7.2	16.0	10.2	11.4	b,c
New Hampshire	10.5	0.3	32	6.9	16.9	9.8	11.1	b,c
New York	10.4	0.2	68	8.2	15.7	10.0	10.7	b,c
Wisconsin	10.2	0.2	49	6.7	13.7	9.8	10.5	b,c
Washington	10.1	0.2	15	8.1	12.3	9.6	10.6	b,c
Nevada	10.1	0.2	71	6.3	13.8	9.7	10.4	c
Minnesota	10.0	0.5	4	8.8	11.0	9.0	11.0	a,b,c

Molt origin of the greatest proportion of starlings collected in states generally west of ˗90° longitude (i.e. Arizona, California, Colorado, Minnesota, New Mexico, Texas, Oregon) and Wisconsin was assigned to the collection state (Table 2). For example, discriminant function analyses suggested that 65% of starlings collected in winter at a dairy or feedlot in Texas replaced their feathers in Texas during the previous summer (Table 2). Molt origin of most starlings collected in Idaho, Nebraska and Nevada was assigned to the state adjacent to the collection state. In contrast, molt origin of the majority of starlings collected in Iowa, Kansas, Missouri and Washington was not assigned to the collection state and/or the state adjacent to the collection state (Table 2).

**Table 2:** Percent of all European starlings (*N* = 686) collected at dairies and feedlots throughout the U.S. and assigned to a molt-origin state using a cross-validation summary of the discriminant function analysis of feather isotopes (by collection state, left column).

		Percent Classifications of Molt Origin by Collection State																		
Collection State	AZ	CA	CO	IA	ID	IL	KS	MN	MO	NC	NE	NH	NM	NV	NY	OR	TX	VT	WA	WI
Arizona	**28**	5	0	3	0	2	12	2	2	3	0	25	10	0	0	0	2	2	0	5
California	5	**40**	0	5	0	0	10	5	0	5	0	0	10	0	0	10	0	0	0	10
Colorado	0	7	**43**	0	7	2	0	2	0	0	11	7	4	2	0	2	0	11	2	0
Iowa	4	16	0	**8**	0	14	4	4	14	2	0	6	14	0	2	0	0	6	0	6
Idaho	0	0	4	0	**23**	0	0	0	0	0	4	0	0	8	0	42	0	0	19	0
Illinois	5	2	5	2	0	**12**	5	10	17	0	0	7	17	0	0	0	2	5	0	12
Kansas	2	12	2	0	0	5	**7**	2	12	29	2	5	2	0	2	0	2	2	0	10
Minnesota	0	0	0	0	0	25	0	**50**	0	0	0	0	0	0	25	0	0	0	0	0
Missouri	5	5	0	0	0	5	5	5	**11**	16	0	5	26	0	0	0	0	5	0	11
North Carolina	0	0	0	0	0	0	16	4	0	**32**	0	0	0	0	0	0	44	0	0	4
Nebraska	0	8	24	0	16	0	0	12	0	0	**12**	12	4	8	0	0	0	4	0	0
New Hampshire	28	6	0	3	0	0	3	16	3	0	6	**9**	6	3	0	0	0	3	0	13
New Mexico	11	0	0	0	0	0	0	0	0	11	11	0	**67**	0	0	0	0	0	0	0
Nevada	0	3	4	1	18	0	1	3	0	0	11	3	1	**14**	0	18	0	6	14	1
New York	3	10	0	1	0	15	1	24	10	0	1	6	1	1	**1**	0	0	3	0	21
Oregon	0	0	20	0	20	0	0	0	0	0	20	0	0	0	0	**40**	0	0	0	0
Texas	5	0	0	0	0	0	0	0	3	26	0	0	2	0	0	0	**65**	0	0	0
Vermont	0	18	12	6	0	0	0	41	0	0	6	6	6	6	0	0	0	**0**	0	0
Washington	0	0	0	0	0	0	0	0	0	0	7	0	0	60	0	20	0	7	**7**	0
Wisconsin	10	0	2	2	2	8	6	14	4	0	2	2	0	0	10	0	0	6	0	**31**

**Fig 2:**
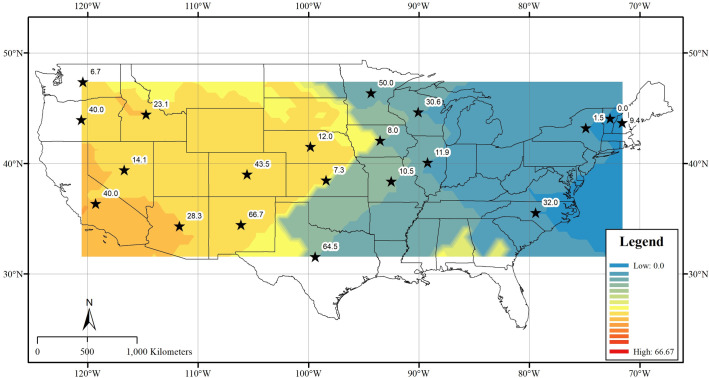
Geographic distribution of collection-state assignments of molt origin among all European starlings (*N* = 686) collected at U.S. dairies and feedlots. For each state associated with this study, the predicted surface represents the log-transformed percentage of samples assigned to the state in which the sample was collected.

Molt origin of the greatest proportion of starlings collected in New Hampshire, New York, North Carolina and Vermont (i.e. east of ˗90° longitude) was not assigned to the collection state and/or the state adjacent to the collection state (Table 2). In contrast, molt origin of the majority of starlings collected in Illinois was assigned to the state adjacent to the collection state. Thus, we discovered a longitudinal gradient of molt origin (e.g. post-molt dispersal) among European starlings collected in winter throughout the U.S. (Fig 2).

### Sex-specific movements

For the purpose of investigating sex-specific origin and movements, we independently replicated our discriminant function analysis with all male and all female starlings. Among all starlings (*N* = 686), 23% were assigned to the collection state and 19% were assigned to the state adjacent to the collection state. For all male starlings (*N* = 489), 23% were assigned to the collection state and 19% were assigned to the state adjacent to the collection state. For all female starlings (*N* = 197), 26% were assigned to the collection state and 13% were assigned to the state adjacent to the collection state. Thus, we observed few differences in the molt origin of male versus female starlings.

### Age-specific movements

For the purpose of testing our prediction that natal dispersal in juvenile starlings would exceed interstate movements in adult starlings collected at U.S. dairies and feedlots, we replicated our discriminant function analysis with 596 adult starlings and then individually assigned molt origin of 90 juvenile starlings based upon upper and lower confidence limits calculated for feather isotopes of all adult samples (Table 3). We observed a greater proportion of juvenile starlings (88%; 79 of 90) assigned to states other than the collection state (i.e. natal dispersal) than that proportion in adult starlings (76%; 451 of 596). Interestingly, 65 of 90 juvenile starlings were collected west of ˗90° longitude and only three of 11 collection-state assignments were juvenile females.

**Table 3:** Interstate comparisons of feather isotopes in 596 adult European starlings collected at U.S. dairies and feedlots. For each of *δ*^2^H, *δ*^13^C and *δ*^15^N, statistically different means between collection states are indicated by unique letters in the right column.

Isotope/state	Mean	SE	*n*	Min.	Max.	Lower CL	Upper CL	Tukey-Kramer *P* < 0.05
*δ*^2^H								
Texas	-43.1	1.1	59	-67.4	-21.6	-45.4	-40.9	a
North Carolina	-52.5	2.1	24	-84.4	-38.8	-56.7	-48.3	a,b
New Mexico	-65.0	4.3	9	-97.2	-53.7	-73.6	-56.4	b,c
Missouri	-65.3	2.8	18	-91.2	-52.2	-70.9	-59.7	c
Kansas	-65.9	2.3	33	-92.4	-42.7	-70.6	-61.2	c
Illinois	-68.7	1.7	31	-86.1	-49.5	-72.1	-65.3	c
Arizona	-70.9	1.2	49	-89.6	-54.9	-73.3	-68.4	c
Iowa	-72.2	1.1	46	-84.7	-56.6	-74.3	-70.0	c
New Hampshire	-76.3	1.8	30	-101.2	-58.8	-79.8	-72.8	c,d
New York	-76.6	1.1	59	-97.5	-60.8	-78.7	-74.5	c,d
Wisconsin	-76.9	1.4	44	-109.9	-53.9	-79.7	-74.0	c,d
California	-79.8	4.7	19	-146.8	-53.5	-89.3	-70.4	c,d
Minnesota	-84.7	4.1	4	-93.1	-75.8	-92.8	-76.6	c,d
Vermont	-86.5	2.3	17	-104.9	-68.4	-91.1	-81.9	d
Colorado	-94.3	1.9	32	-112.9	-70.3	-98.1	-90.4	d
Nebraska	-96.0	3.1	20	-121.8	-64.4	-102.1	-89.8	d
Nevada	-107.5	1.9	68	-139.9	-64.3	-111.2	-103.8	e
Washington	-108.3	3.5	14	-133.7	-88.3	-115.3	-101.3	e
Oregon	-115.1	9.9	4	-135.0	-94.7	-134.9	-95.2	e
Idaho	-115.2	2.1	16	-128.3	-98.1	-119.3	-111.0	e
*δ*^13^C								
Texas	-17.3	0.4	59	-22.1	-11.1	-18.1	-16.5	a
California	-19.0	0.6	19	-24.5	-14.3	-20.3	-17.7	a,b
New Mexico	-19.2	0.5	9	-22.5	-17.0	-20.3	-18.2	a,b,c
North Carolina	-19.4	0.5	24	-24.1	-15.8	-20.4	-18.4	b
Arizona	-19.5	0.3	49	-22.6	-12.7	-20.1	-19.0	b
New Hampshire	-19.9	0.6	30	-24.0	-12.3	-21.1	-18.8	b,c
Kansas	-20.8	0.4	33	-23.9	-15.0	-21.6	-20.1	b,c
Iowa	-21.0	0.3	46	-24.0	-14.9	-21.7	-20.4	b,c
Missouri	-21.2	0.4	18	-23.7	-17.8	-22.1	-20.4	b,c
Illinois	-21.7	0.4	31	-24.7	-16.7	-22.5	-21.0	c
Vermont	-21.8	0.6	17	-24.8	-16.5	-23.0	-20.5	c
Colorado	-21.8	0.4	32	-26.0	-16.2	-22.5	-21.0	c
Oregon	-21.9	0.8	4	-23.5	-19.8	-23.4	-20.3	b,c
Washington	-22.0	0.2	14	-24.1	-20.9	-22.5	-21.5	c
Nevada	-22.2	0.2	68	-25.7	-12.7	-22.6	-21.7	c
New York	-22.2	0.3	59	-25.1	-16.0	-22.8	-21.6	c
Wisconsin	-22.4	0.2	44	-25.0	-18.5	-22.8	-22.0	c
Nebraska	-22.4	0.5	20	-25.8	-15.7	-23.4	-21.5	c
Idaho	-22.7	0.2	16	-23.6	-21.1	-23.0	-22.4	c
Minnesota	-23.6	0.3	4	-24.1	-22.8	-24.2	-22.9	c
*δ*^15^N								
Texas	13.6	0.4	59	9.2	24.3	12.9	14.3	a
New Mexico	13.4	0.6	9	10.2	16.9	12.2	14.6	a
Colorado	12.9	0.4	32	9.7	17.3	12.1	13.7	a
California	12.6	0.6	19	6.7	17.2	11.4	13.8	a
North Carolina	11.6	0.6	24	6.8	16.6	10.3	12.9	b
Illinois	11.5	0.5	31	8.1	18.2	10.5	12.5	b
Iowa	11.5	0.3	46	8.1	15.5	10.9	12.0	b
Vermont	11.4	0.5	17	8.3	14.7	10.4	12.3	b
Missouri	11.4	0.5	18	8.2	14.5	10.4	12.3	b
Oregon	11.1	1.0	4	9.6	13.8	9.2	13.0	a,b
Arizona	11.1	0.2	49	7.8	16.2	10.6	11.6	b
Idaho	10.9	0.4	16	8.5	13.9	10.1	11.6	b
Kansas	10.8	0.4	33	7.2	16.0	10.1	11.5	b
Nebraska	10.8	0.6	20	6.5	15.1	9.7	12.0	b
New Hampshire	10.5	0.3	30	6.9	16.9	9.8	11.2	b
New York	10.4	0.2	59	8.3	15.7	10.0	10.8	b
Washington	10.2	0.3	14	8.1	12.3	9.6	10.7	b
Wisconsin	10.1	0.2	44	8.0	12.3	9.7	10.4	b
Minnesota	10.0	0.5	4	8.8	11.0	9.0	11.0	a,b
Nevada	10.0	0.2	68	6.3	13.8	9.6	10.4	b

### Relationship of predicted precipitation *δ*^2^H and feather *δ*^2^H

**Fig 3:**
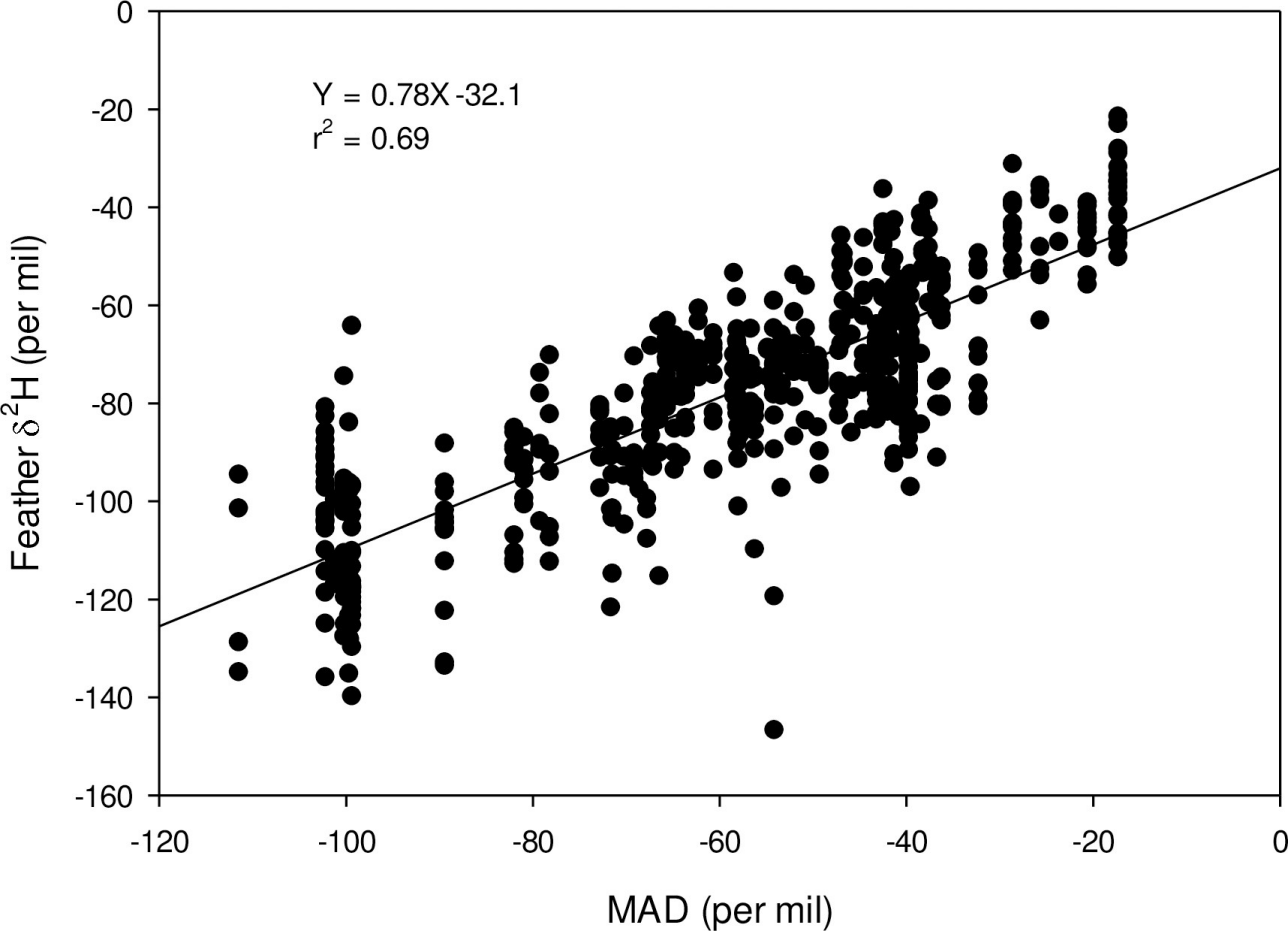
Relationship between feather δ^2^H and amount-weighted mean annual precipitation δ^2^H (MAD) for 596 adult starlings collected across the United States.

For adult starlings, we found a strong relationship between feather δ^2^H and the amount-weighted mean-annual precipitation δ^2^H (δ^2^H_f_ = 0.78* δ^2^H_p_ -32.1; residual SD = 8.5‰; r^2^ = 0.69; Fig 3) which closely agreed with the relationship between feather δ^2^H and the amount-weighted mean growing-season precipitation δ^2^H (δ^2^H_f_ = 0.83* δ^2^H_p_ -32.4; residual SD = 9.7‰; r^2^ = 0.68; not shown). No differences were found between sexes for adult starlings for both of these correlations. For juvenile starlings, the correlation between feather δ^2^H and the amount-weighted mean-annual precipitation δ^2^H (δ^2^H_f_ = 0.64* δ^2^H_p_ -44.7; residual SD = 10.17‰; r^2^ = 0.55) was close to that found for amount weighted growing-season precipitation δ^2^H (δ^2^H_f_ = 0.69* δ^2^H_p_ -44.9; residual SD = 8.8‰; r^2^ = 0.54).

### Relationship of *δ*^13^C and *δ*^15^N values with agricultural crops

**Fig 4:**
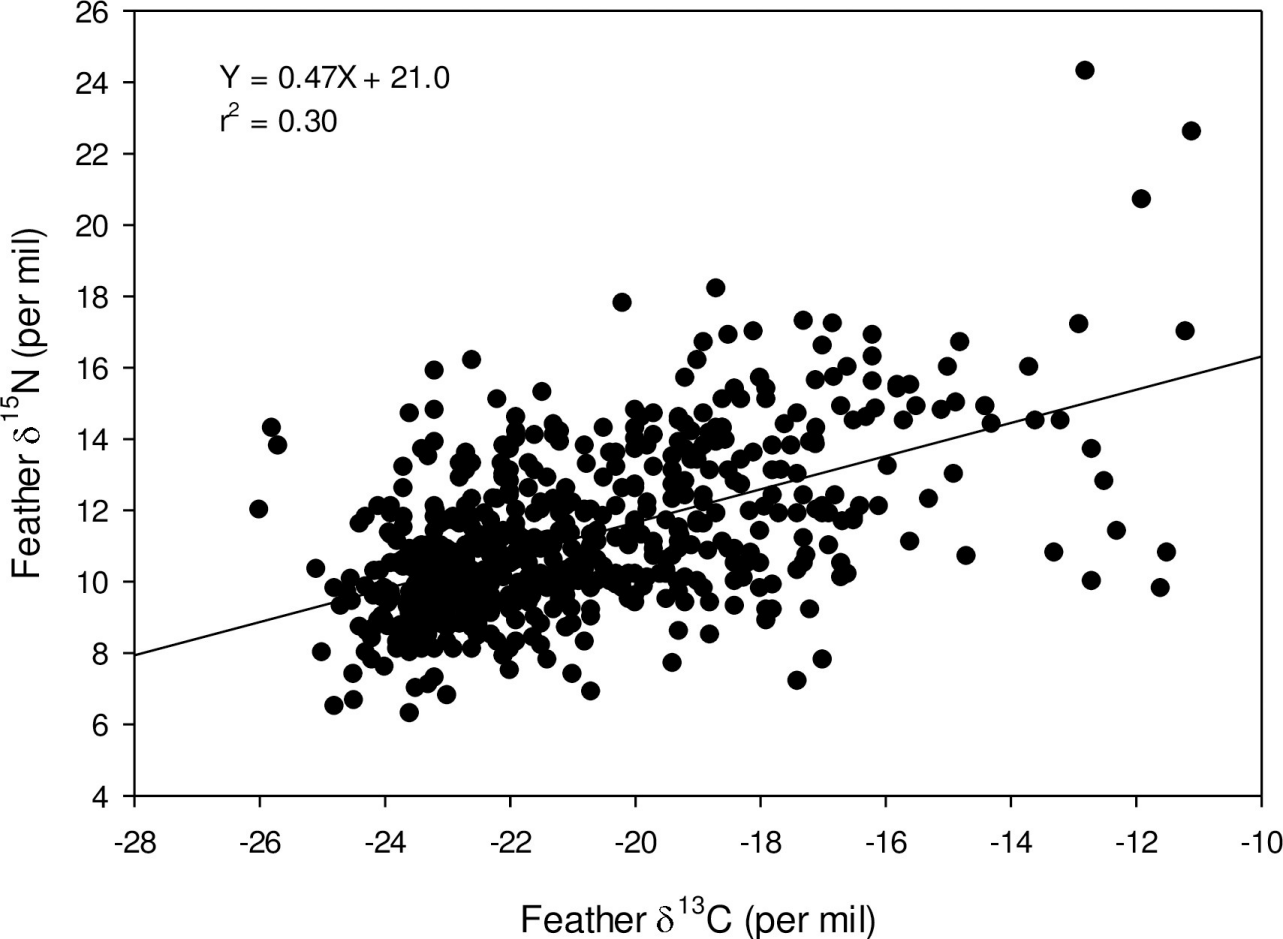
Relationship between feather δ^15^N and δ^13^C for 596 adult starlings collected across the Unites States.

The predicted percent C4 contribution to starling diets varied among states and age groups (Table 4). Adult C4 contribution ranged from 16.4% in Minnesota to 56.1% in Texas. For juveniles, the range was from 10.7% in New Hampshire to 43.6% in Texas. We found a positive relationship between feather δ^15^N and δ^13^C for adult starlings (Fig 4) but not for juvenile starlings (r^2^ = 0.02).

**Table 4:** Summary of derived percent C4 in diets of European starlings by collection state and age group. The model was based upon a simple two endpoint expectation of a 100% C3 diet producing a feather δ^13^C value of -26.0‰ and that of a 100% C4 diet producing a feather δ^13^C value of -11.0‰ (after [1]).

	Adult Starlings (*N* = 596)			Juvenile Starlings (*N* = 90)		
Collection State	δ^13^C (‰)	*n*	% C4	δ^13^C (‰)	*n*	% C4
Arizona	-19.5±1.9	49	43.1±12.7	-20.1 ±1.1	11	39.3±7.4
California	-19.0±2.8	19	46.6±18.7	-21.8	1	28.1
Colorado	-21.8±2.2	32	28.3±14.3	-22.1±1.2	14	26.2±8.3
Idaho	-22.7±0.7	16	21.9±4.3	-23.0±0.9	10	20.2±5.8
Illinois	-21.7±2.2	31	28.5±14.3	-22.0±2.1	11	27.0±13.6
Iowa	-22.1±2.1	46	33.1±14.2	-21.2±2.1	4	31.9±13.9
Kansas	-20.8±2.2	33	34.5±14.3	-20.2±2.9	8	38.7±19.5
Minnesota	-23.6±0.6	4	16.4±4.0			
Missouri	-21.2±1.8	18	31.9±11.9	-23.3	1	18
Nebraska	-22.4±2.2	20	23.7±14.3	-19.9±4.1	5	40.5±27.0
Nevada	-22.2±1.9	68	25.7±13.2	-19.9±1.7	3	40.7±11.4
New Hampshire	-19.9±3.3	30	37.5±20.8	-24.4	2	10.7
New Mexico	-19.2±1.5	9	45.3±3.5			
New York	-22.2±2.3	59	25.4±15.1	-22.5±2.3	9	23.5±15.4
North Carolina	-19.4±2.5	24	44.0±16.7	-22.3	1	24.7
Oregon	-21.9±1.6	4	27.7±10.5	-23.4	1	17.3
Texas	-17.6±3.0	59	56.1±19.9	-19.5±3.7	3	43.6±24.3
Vermont	-21.7±2.5	17	28.2±17.6			
Washington	-22.0±0.8	14	26.6±6.1	-21.9	1	27.2
Wisconsin	-22.4±1.4	44	24.0±9.5	-21.4±1.5	5	30.5±10.1

## Discussion

Our extensive isotopic analyses of the feathers of adult and juvenile starlings collected in winter at dairies and feedlots across the U.S. revealed considerable isotopic variability associated with movements among states. These results underline the utility of the isotope approach to investigate movement patterns of such introduced species, especially within context of management approaches [18]. We predicted that starlings collected south of 40° latitude would primarily be assigned to the collection state because starling populations south of 40° latitude exhibited little migration in winter away from their nesting location [22]. Among collection states south of 40° latitude, the greatest proportion of starlings collected in Arizona, California, Colorado, New Mexico and Texas were assigned to the collection state. In contrast to our prediction, the greatest proportion of starlings collected in Minnesota, Oregon and Wisconsin were also assigned to the collection state; these collection states are each north of 40° latitude. Also in contrast to our prediction, the greatest proportion of starlings collected in Kansas, Missouri and North Carolina were not assigned to the collection state; these collection states are each south of 40° latitude. We therefore observed varied molt origins and interstate movements throughout the U.S. and these movements were not exclusively predicted by the latitude of our collection states.

Interestingly, molt origins of starlings collected at dairies and feedlots generally west of ˗90° longitude (i.e. 11 of 15 states west of the Mississippi River, including Wisconsin) were assigned to the collection state and/or the state adjacent to the collection state (Table 2). In contrast, molt origin of starlings collected generally east of ˗90° longitude (four of five eastern states) was not assigned to the collection state and/or the state adjacent to the collection state. We therefore observed a longitudinal pattern in predicted molt origin of European starlings collected at U.S. dairies and feedlots in winter.

We also predicted that natal dispersal in juvenile starlings would exceed interstate movements in adult starlings collected at U.S. dairies and feedlots. We observed a greater proportion of juvenile starlings (*N* = 90) assigned to states other than the collection state (i.e. likely due to migration or natal dispersal) than that proportion in adult starlings (*N* = 596). Our isotopic findings are consistent with results of banding studies designed to examine breeding and natal dispersal in starlings. For example, breeding dispersal distances in AHY-banded starlings was 15 ± 5 km in males (± SE; *N* = 99) and 10 ± 4 km in females (*N* = 109) in northeastern U.S. [22]. In contrast, natal dispersal distances in hatching-year (HY)-banded starlings was 80 ± 10 km (*N* = 400 males and females) [22]. Approximately 30% of 468 starlings banded as nestlings or fledglings were recovered in subsequent breeding seasons > 50 km from their natal nest location and approximately 12% of these HY-banded starlings dispersed distances > 200 km [22]. We therefore observed high dispersal rates, particularly in juvenile starlings as previously reported [14, 21].

We found a strong relationship between mean annual and growing season precipitation at collection sites and feather δ^2^H for adult starlings (r^2^ = 0.69). This relationship with a variance estimate of the residuals as 8.5‰ is among the lowest reported for various species of known origin in North America [28] and provides strong evidence for overall site fidelity among adult starlings compared to juvenile starlings which showed a poorer relationship (r^2^ = 0.55) and higher residual variance (8.8 to 10.2‰). We consider the results from this single isotope measurement to be encouraging but recognize that the discriminant function analysis approach used here for assignment of birds to state to be more appropriate given the fact that starlings consumed cattle and dairy cow foods which were non-natural and likely not local.

Estimates of C4 carbon input into adult starling diets during feather growth ranged from mean values of 16.4% (New Hampshire) to 56.1% (Texas), and 20.2% (Idaho) to 43.6% (Texas) for juvenile starlings. We did not measure isotopically foods from sites across the U.S. and so it is not clear if these values reflect difference in the composition of cow and cattle foods that may have been consumed during the feather growth period. As well, there is evidence that starlings consume selected fractions from feeds provisioned to livestock [12]. Nonetheless, our results provide evidence that stable isotopes could be used in the future to estimate contributions of cow and cattle feed vs other prey items to diets of starlings. The weak but positive relationship between feather δ^15^N and δ^13^C suggests that more corn in the diet was associated with higher δ^15^N possibly the result of greater use of nitrogen-based fertilizer in corn production and subsequent enrichment in ^15^N due to ammonification.

Our study provides a foundation for future applications of multi-isotopic tracing of molt origin, and future inquiries regarding the ecology and movements of migratory birds in agroecosystems. Future uses of our state-specific assignments of molt origin may include management applications and ecological research designed to test hypotheses regarding the origin and movements of European starlings. For example, we used our existing feather isotopes database to determine molt origin of locally-overabundant starlings at a Montana dairy. Although the dairy manager generally observes starlings at this site throughout the year, they wondered if their wintering starlings originated from Montana. USDA’s Wildlife Services professionals collected ten starlings from this site in January 2018. We again used discriminant function analysis to assign states of molt origin based upon *δ*^2^H, *δ*^13^C and *δ*^15^N values in these collected feather samples. The origin of five, three and two of these starlings collected in Montana was assigned to Montana, Idaho and Oregon, respectively. Thus, feather isotope analyses and our multi-isotopic data can be used to (1) develop our fundamental understanding of European starlings associated with U.S. dairies and feedlots and (2) inform efficacy of control measures based upon the origin and movements of starlings at these sites.

### Future research

An optimal approach to investigating origins of migratory wildlife using stable isotopes would include ground-truthed isotopic basemaps or isoscapes based upon same-year measurements of breeding and wintering subpopulations [29]. The proportion of local versus immigrant birds in wintering flocks can be investigated for the development of strategies designed for management of pest birds in agroecosystems. In addition, there is potential to examine how flock composition (e.g. age structure, immigrants, residents) may change through time and in response to management prescriptions on breeding and wintering grounds [1].

The collection and isotopic analyses of specific age classes can be used to determine breeding status, fidelity and movements of migratory birds. The paucity of breeding status and age class information is a primary limitation of North American banding data [3]. Feather isotopes can also be used to investigate dispersal and migration of first-year versus second-year breeders, and associated nest- and wintering-site fidelity. Specifically, feather isotopes can be used to test the hypothesis that second-year breeders do not develop an attachment to an area and thus their breeding dispersal is like that of natal dispersal [3]. Moreover, feather isotopes can be used to investigate biotic (e.g. human and bird densities, winter-roost communities) and abiotic (climate, water availability, land use, habitat dynamics) influences on seasonal and regional movements of European starlings and other migratory birds.

More broadly, isotopic investigations can be used to test migratory trends, and relevant predictions regarding 1) starlings may be more migratory on the edge of their range or within low-density populations and 2) areas with highest out-of-state banding recoveries (or molt assignments to other than collection states) are close to main flyways of starling migration [3]. In context of bird damage management, stable isotopes can be used to investigate if starling populations exhibiting high natal dispersal are likely to reinvade low density areas with reliable food sources (e.g. dairies or feedlots using non-lethal or lethal control) at a much faster rate than blackbird species that exhibit little difference in natal and breeding dispersal distances [22].

We recognize that isoscapes may not necessarily conform to assignment of individuals to within state boundaries. We opportunistically collected European starlings during winter in the 20 states most commonly associated with starling damage management provided by USDA’s Wildlife Services program and at U.S. dairies and feedlots that experience starling damages. Thus, we sampled starlings within states (i.e. populations) of interest and then developed relevant inferences about starlings in those sampled states. Previous studies have used a more spatially explicit probabilistic assignment of individuals to geographic origins [17, 23]. As such, the analyses we performed based on the DFA approach may have underestimated the amount of dispersal among states. On the other hand, the DFA assignment approach did allow us to use a three isotope approach vs. the single isotope (δ^2^H) approach because only the precipitation-based feather δ^2^H isoscape is currently available for this purpose. The DFA approach did rely on the assumption that most sampled birds originated from their collection state. In general, we recommend multi-isotopic approaches to ecological investigations, including wildlife response to management actions and environmental change, vertebrate pest-agronomic associations, post-molt dispersal, and the biology of juvenile and adult starlings [1, 27]. Ideally, feathers from known molt origin birds could be used to construct starling-specific feather isoscapes. Field efficacy trials of lethal and non-lethal management actions can be enhanced by understanding movements of starlings following starling damage management at U.S. dairies and feedlots [30]. Similarly, investigations regarding net impacts of pest birds to plant and animal agriculture, feeding ecology during the nonbreeding season and aspects of physiology related to migration can be enhanced by an understanding of the movements and habits of these birds at appropriate spatial scales [1].

## Supporting information

S1 Table Werner SJ, Fischer JW, Hobson KA. Multi-Isotopic (*δ*^2^H, *δ*^13^C, *δ*^15^N) Tracing of Molt Origin for European Starlings Associated with U.S. Dairies and Feedlots. Figshare 2020; https://dx.doi.org/10.6084/m9.figshare.12652913.
(XLSX)
